# Development of a new kappa-carrageenan hydrogel system to study benthic diatom vertical movements

**DOI:** 10.1371/journal.pone.0297962

**Published:** 2024-04-11

**Authors:** Arianna Rizzo, Alessandro Ajò, Huixuan Kang, Luisa De Cola, Bruno Jesus

**Affiliations:** 1 Institut des Substances et Organismes de la Mer–ISOMer UR 2160, Faculté des Sciences et des Techniques, Nantes University, Nantes, France; 2 Department of Molecular Biochemistry and Pharmacology, Istituto di Ricerche Farmacologiche Mario Negri, IRCCS, Milano, Italy; 3 Pharmaceutical Science Department, University of Milan, Milan, Italy; King Abdulaziz University, SAUDI ARABIA

## Abstract

Benthic diatom vertical movement has been investigated mainly through indirect measurements based on chlorophyll *a* fluorescence and spectral reflectance signals. The presence of sediment hinders direct imaging and grazers activity renders the work under controlled conditions very difficult. This study provides a tool to study diatoms movement in a 3D hydrogel matrix. Synthetic and natural hydrogels were tested to find the best 3D transparent scaffold where diatoms could grow and freely move in all directions. Polyamidoamines (PAAm) hydrogels were no-cytocompatible and hyaluronic acid (HA) only allowed diatoms to survive for 2-days. Natural hydrogels made of gelatin/Na-alginate, Na-alginate and kappa-carrageenan (KC) were cytocompatible, with KC showing the best properties for diatom growth and movement on a long term (up to 2 months). Comparing *Nitzschia spathulata*, *Gyrosigma limosum* and *Navicula phyllepta* growth in liquid media vs in KC gels, we found that diatoms reached a significantly higher final biomass in the hydrogel condition. Hydrogels were also useful to isolate large size diatom species e.g., *Nitzschia elongata*, that did not survive in suspension. Finally, we showed three ways to study diatom species-specific movement in KC hydrogels: 1) controlled species mix; 2) natural diatom assemblages with grazers; and 3) natural diatom assemblages without grazers. With our system, single diatoms could be imaged, identified, and counted. In addition, different stimuli, e.g., light intensity and light composition can be applied and their effects on movement and physiology studied without being masked by sediment or impaired by meiofauna.

## 1. Introduction

Benthic diatoms found at the water-sediment and air-sediment interface in intertidal mudflats form with cyanobacteria and euglenoids, a community immersed in a mucilage matrix, called microphytobenthos (MPB) [1, 2]. MPB hosts a rich variety of motile pennate diatoms, which colonize muddy sediments (epipelon) [3–5]. During diurnal low tides MPB forms transient biofilms at the sediment surface [6]. These benthic motile diatoms exhibit two photoprotection mechanisms to cope with the strong variable light conditions typical of mudflats. The first mechanism is a physiological photoprotection, regulated by the xanthophyll pigment cycle and associated non-photochemical quenching [7, 8]. The second is a behavioural response, triggered by light intensity and tidal rhythms, consisting in vertical movements used by cells to position themselves at the sediment depth that optimizes their photosynthetic rates [9, 10]. Recently, it has been proposed that cell orientation within the biofilms might also work as a photoregulatory mechanism [11]. Most of the studies have used freshly collected MPB and biomass proxy measurements to follow the vertical movements, e.g. spectral reflectance analysis using normalized difference vegetation index (NDVI) or pulse amplitude modulated (PAM) fluorometry using the minimum fluorescence yield parameter (*F*_*o*_) [4, 12, 13]. Working with fresh MPB has the advantage of using cells at their best physiological state and photoacclimated to environmental light conditions. Natural MPB biofilms also keep their endogenous rhythm, which is maintained in laboratory conditions for few days, allowing observations close to *in-situ* conditions [6, 14]. However, following diatom movements and species-specific diatom responses in natural environments is challenging due to the presence of sediment and non-controlled elements e.g., presence of grazers and other microalgal groups that might change the MPB composition after few days from collection [15, 16]. The difficulty in assigning the fluorescence signal to different diatoms species in the MPB has been addressed in previous studies identifying the migrated species by LTSEM [17–19] or by coupling the reflectance signal with collecting diatoms at their maximum migration peak using the tissue lens method for their identification [11, 17, 18]. A list of the most used methods to identify the MPB is available in [19], where authors also assess the limit of the above techniques in reconstructing the 3D structure of the MPB.

Using optical measurements to study MPB directly on collected sediments is challenging. Muddy sediments are optically dense, exhibiting very high light attenuation coefficients 1.5–2.5 mm^-1^ up to 3.42 rendering optical microscopy observations very difficult [20]. Working with a controlled optically transparent setup would allow to identify the diatoms present in the assemblage, to determine the depth at which they move and how individual cells change their orientation in response to light stimuli. Also, there is species-specific variation in migration patterns [7, 12, 19, 21–23], which can be better observed in a controlled transparent system than in sediment, such as transparent hydrogels, where diatoms could attach and glide within a porous 3D network.

Overall, hydrogels are defined as hydrophilic three-dimensional porous networks, composed by polymeric chains cross-linked by covalent or non-covalent bonds, which can retain high amount of water without dissolution [24, 25]. To the best of our knowledge, hydrogels have never been used as scaffold to study MPB movements. However, embedding diatoms in hydrogels without impacting their physiological status, their capacity of moving freely while making direct optical observations of the system, requires a careful selection of the hydrogel to use.

A suitable hydrogel needs to be: cytocompatible, transparent, high in water content, porous enough to allow diatom movement inside, sufficiently stiff and stable to allow measurements to be carried out over time. Hydrogel transparency is essential for monitoring algal growth and tracking movement in transmittance and to allow light penetration. High water content is a hydrogel feature, which can reach up to 99% of water content [26]. High water content is necessary for nutrient circulation, for algal cells osmolarity and for reproducing the sediment high water content. These features are also dependent on porosity, defined as the amount of pore spaces present in the hydrogels [27]. Stiffness is important for gel integrity over time, to support cell growth and proliferation inside the hydrogel, and to allow diatoms digging their way inside the 3D scaffold. Hydrogel stability over time is important to carry out experiments during the entire diatom growth curve, i.e., 15 days minimum as a common threshold to low and high growth rate species [28, 29]. Hydrogel stability also depends on the ionic strength, since the presence of cations, like in seawater, can modify the hydrogel features [30]. Another important property is bioadhesion which refers to the capability of a biological material to adhere to a surface [31]. This needs to be adjusted to allow cell adhesion to the hydrogel surface without impeding cell movement and the formation of cell aggregates.

The aim of this study was to develop a suitable hydrogel setup for monitoring epipelic diatom movement according to the above-mentioned criteria. We looked for a transparent scaffold, convenient for imaging techniques that would allow to assess diatoms orientation and position in 3D, and to afterwards correlate these findings with diatoms physiology.

## 2. Materials and methods

Seven different hydrogels were fabricated, for all we tested their cytocompatibility and measured their storage modulus and water content. Transparency was measured only on the cytocompatible hydrogels. Diatoms were inoculated from the top or in the hydrogel mix before polymerization when diatoms vitality was not compromised by polymerization. Once the best hydrogel was selected, diatoms growth in liquid media (the most common method) and in hydrogels was compared. Finally, two methods to transfer fresh MPB onto the hydrogel were tested.

### 2.1 Diatom collection and maintenance

Diatom species were isolated from the Bouin mudflat (Bourgneuf Bay, France, 47.015753, 2.024148) during low tide. The first half centimetre of the sediment was collected by scrapping and transported back to the laboratory where it was spread down in trays and covered with water from the same site. Trays were kept under natural illumination and the sediment allowed to settle down until the next day when it was drained and diatoms collected by letting them migrate upwards into lens tissues placed at the sediment surface [17]. The lens tissues were then immersed in filtered seawater and diatoms resuspended by agitation in filtered sea water from the site.

Three diatom species were selected to test hydrogels toxicity: *Gyrosigma limosum*, *Nitzschia spathulata and Navicula phyllepta*. The species selection was done considering their motility and their intrinsic characteristics useful for the screening: *G*. *limosum* is easy to track due to its large size (100 μm); *N*. *spathulata* grows fast (μ_max_ = 1.32) is 300 μm large and survives in sub-optimal conditions in laboratory conditions; *N*. *phyllepta* is from the genus *Navicula* which is very common among epipelic diatoms. These species were isolated with the single cell picking technique and acclimated to laboratory conditions for seven months in F/2 medium under a 16/8 hour light/dark regime at 20° C, at 100 μmol photon m^-2^ s^-1^, without shaking or aeration. In *Nitzschia spathulata* isolation, the single isolated cell usually died in suspension without dividing, thus the single cell was instead transferred into a 1.5% KC hydrogel polymerized in a 24 well-plate. Once *N*. *spathulata* cells divided, the gel was transferred to a 100 mL Erlenmeyer with F/2 medium and started to attach to the flask’s bottom, forming biofilms. Cultures were kept in exponential phase by frequent reinoculation, every 2 weeks.

### 2.2 Hydrogels fabrication

Seven different hydrogels were tested based on published microalgae applications [32–35] and on manufacturers’ safety data sheet toxicity information. The main requirement for the first screening was hydrogel cytocompatibility. All hydrogels were first prepared in a transparent vial for reverse testing and then 400 μL were poured in 24-well plates in 5 replicates.

#### 2.2.1 Polyamidoamines hydrogel

Polyamidoamines (PAAm) hydrogels were prepared accordingly to method [36]. Briefly, 200 mg N,N’-methylenebisacrylamide (MBA) and 50 mg γ-aminobutyric acid (GABA) were mixed with 1.5 mL of milliQ water (magnetic stirring at 37°C); 75 mg of pentaethylenhexamine (PEHA) were then added and after complete homogenization the solution was poured in 24-well plates. After 45 minutes, the solution switched from turbid to clear and after 3 hours the sol-gel transition happened. Removing PAAm hydrogel toxicity was attempted with two methods: 1) rinsing four times with distilled water; and 2) by lyophilization and re-hydration to make the hydrogel inert and use its structure as a scaffold.

#### 2.2.2 Polyamidoamines hydrogel with alginate

Six ml of milliQ water was mixed with 120 mg of sodium alginate and 315 μL of PEHA and once well mixed and homogenized it was further mixed with 1 g of MBA and 250 mg of GABA. After complete homogenization the solution was poured in 24-well plates. Gelation occurred after 1 hour.

#### 2.2.3 Sodium silicate hydrogel

Sodium Silicate hydrogel was made by mixing 1300 μL of milliQ water with 411 μL of Na-silicate (10% w/v NaOH and 27% w/v SiO_2_). We tested gelation by adding 4N HCl from 150 μL up to 200 μL, with 10 μL increases, until gelation occurred. pH was measured with pH test strips; after HCl addition, pH lowered from 12 to 8 leading to hydrogel formation and a final pH biocompatible with diatoms [37]. The gel was formed in a few seconds after the addition of HCl, therefore it was prepared directly in 24-well plates.

#### 2.2.4 Hyaluronic acid hydrogel

Methacrylated hyaluronic acid (MeHA) hydrogel was used to crosslink the hydrogel with the dithiothreitol (DTT) used as the reaction initiator. MeHA solution was prepared using two different HA molecular weight (470 KDa and 1700 KDa) as following: 20 mg of met-HA 470/1700 KDa were dissolved in 953/850 μL of phosphate-buffered saline (PBS) overnight. 7 mg DTT were dissolved in 100μL of PBS. 47.1 μL of this DTT solution were added to the mix. After complete homogenization, solution was poured in 24-well plates.

#### 2.2.5 Na-alginate

Na-alginate (Sigma-Aldrich Co, viscosity 4000 cps) polymerization is caused by ionic cross-linking reaction with 1% w/v CaCl_2_ solution. Higher CaCl_2_ concentration will induce higher dehydration, which might potentially hamper cell motility inside the gel. After testing different Na-alginate concentrations (1.5%, 2%, 2.5%, 3%, 3.5% w/v), the 2% concentration was selected as the most suitable, since at 1.5% the hydrogel degraded in seawater in 7 days, while at higher concentration diatom movement was slowed down due to the mechanical confinement of the polymeric network. To obtain a cylindrical shaped Na-alginate hydrogel, firstly, 1% w/v agar solution was heated at 100° C before being poured into 24-well plates and polymerized at room temperature. The polymerized agar functioned as a template for the Na-alginate hydrogel. Therefore, 300 μL 1% w/v CaCl_2_ was then spread on the top of the polymerized agar and left for evaporation under a sterile hood for one hour. Once all the liquid had evaporated and the CaCl_2_ absorbed by the gel, 400 μL of 2% Na-alginate solution was poured and left to polymerize for 12 hours. To avoid bacteria contamination before the algae inoculum, the hydrogel was UV-sterilized (Philips, UV-C, 30W) for 20 minutes. The hydrogel was maintained hydrated with F/2 medium addition.

#### 2.2.6 Kappa carrageenan (KC)

1.5g Kappa carrageenan (Sigma-Aldrich Co, purity > 99%) was dissolved in 100 mL deionized water at 70°C using magnetic stirring, resulting a homogeneous and transparent 1.5% w/v KC solution. The polymerization occurred while the solution was cooled down to room temperature. The gels were then exposed to UV light for 20 mins for sterilization before inoculating the diatoms.

#### 2.2.7 Gelatin hydrogels

Different amount of gelatin powder (Sigma Aldrich, type B) was dissolved into deionized water at 50°C under magnetic stirring, resulting in 2%, 4%, 6%, 8%, 10% w/v gelatin solution. The gelatin hydrogels were formed by physical crosslinking in water above a certain concentration at room temperature. Different gelatin concentrations were tested to identify the best for polymerization; 2% and 4% did not polymerize; 6%, 8% and 10% were selected due to the high stability, fast gelation, and desired stiffness for cell motility. However, since the strong bioadhesion property of the gelatin might hinder the movement of diatoms, a new gel of gelatin mixed with Na-alginate was formulated to decrease gelatin adhesion [38]. Gelatin at 8% w/v was the minimum concentration required for the polymerization with 1.5% w/v Na-alginate. The newly formulated 1.5% Na-alginate/8% gelatin hydrogel was tested in 3 different ratios: 3:1, 2:1 and 1:2.

### 2.3 Hydrogels characterization

Stiffness is expressed as the storage modulus (G’) and it is measured in Pascal. Storage modulus (G’) was measured using Discovery HR-2 rheometer (TA instruments) equipped with an 8 mm parallel plate (with 1.0 mm zero gap) measuring system at room temperature. After the formation of hydrogels, a strain-sweep test over 0.1 to 10% strain at a fixed oscillatory frequency of 1.0 Hz was performed to identify the storage modulus. Each sample was measured in triplicate.

The equilibrium water content (EWC) was measured as:

$$EWC=\frac{W_{\mathrm{wet}}‐W_{\mathrm{dry}}}{W_{\mathrm{wet}}}x100$$

where W_wet_ and W_dry_ represent the mass of the hydrogel sample after and before swelling.

EWC was determined by swelling the crosslinked hydrogels in deionized water for 1 hour at room temperature. The gels were then removed and the water in excess absorbed with lens tissue paper. Finally, the W_wet_ of the swollen gels was determined by an analytical balance (Sartorius). The hydrogels were afterwards lyophilized and W_dry_ measured.

Transparency was measured on gelatin/Na-alginate, Na-alginate and KC hydrogels. Gel transparency was measured by transmittance at 600 nm using an integrating sphere (Labsphere) connected to a spectrometer (Avantes AvaSpec-ULS-RS-TEC) and a Xenon lamp (Ocean Optic), coupled via two 1 mm core fibres (FC-1000-2-SR, Avantes) to the sphere from the light source, and from the sphere to the spectrometer. The light source was warmed up for one hour before use, to allow stabilization. All measurements were run in a dark room. The background signal was acquired with no illumination light and the reference signal with light on but no sample.

### 2.4 Diatoms inoculum inside the hydrogels

Approximately 1000 cells inoculum (20 μL) of *N*. *spathulata*, *G*.*limosum*, *N*.*phyllepta* were added to the hydrogels. A non-motile species, *Odontella aurita*, was used as a control to ensure the presence of diatoms inside the hydrogel was due to an active movement towards the bottom and not to a sedimentation artefact from the presence of big pores in the hydrogel or from leaking. According to the type of polymerization, the inoculum was either added to the gel mixture pre-polymerization (1) or after polymerization by depositing cells on the top (2).

1. In PAAm, PAAm + Na-alginate, hyaluronic acid, gelatin/ Na-alginate and Na-alginate hydrogels, diatoms could be inoculated both before and after the gel polymerization. Before polymerization, the inoculum was added to the liquid mix at room temperature (20°C), letting the solutions cool down at ambient temperature. Inoculum size was kept low (20 μL) to avoid altering the gel composition and final volumes. The solution was gently mixed by hand to avoid diatom breakage. The inoculum addition time changed according to the hydrogel: in PAAm diatoms were added soon after PEHA addition. In the Hyaluronic acid hydrogel, diatoms were inoculated after the DTT addition. In the Na-alginate and gelatin gel, the algae were added in the Na-alginate mixture before mixing with gelatin. In the Na-alginate gel, diatoms were added to the solubilized Na-alginate solution before the crosslinking with the CaCl_2_.
2. In the sodium silicate and the KC hydrogels the inoculum was added on the hydrogel top after gelation. Sodium silicate solution has a basic pH (12) and the KC solubilizes at high temperature, 80°C, both conditions would be lethal for diatoms if added during the polymerization phase. In this case, a 100 μL inoculum with all the 4 species mentioned, was poured and spread on the hydrogel’s surface, after polymerization.

Diatoms were left to attach to the hydrogels surfaces for 30 minutes under the sterile hood and their adhesion was checked by visual observation under the microscope (Olympus CH40, 100X). 200 μL of fresh F/2 medium was added to cover the cell-attached hydrogels and prevent water evaporation. Cells viability was afterwards checked at the optical microscope, looking at the cell integrity and motility. When the hydrogels were too opaque, a fluorescence microscope was employed to visualize diatoms using chloroplast autofluorescence. The presence of organelles, diatom dispersion inside the gel, colonization of the entire volume, hence their movement capacity, were all positive indicators of cells viability. Contrastingly, cells emptied of their chloroplasts were considered dead. Hydrogels with diatoms inside were incubated in 24-well plates with 200 μL of culture medium and kept in a Controlled Temperature (CT) room at 18°C with a 16:8 L/D cycle under 70 μmol photons m^-2^ s^-1^ and checked the following day to assess cells viability. For hydrogels kept more than 2 weeks, 50 μL milliQ addition every 2 days was necessary to compensate for evaporation and 100 μL F/2 medium was added to provide nutrients for the diatoms every 15 days if the system was kept in continuous.

### 2.5 Transference of microphytobenthic diatoms to the hydrogels

To transfer diatoms from MPB to the hydrogels, two methods were employed:

In the first, diatoms were collected with the tissue lens method explained above. The biomass recovered was washed off in a 2L glass container with fresh F/2 medium. To clean diatom species from the grazers populating the sediment, two homogeneous subsamples of 200 mL were taken and one mixed with 50 mg/L of sodium dodecyl sulphate (SDS), and the other without any treatment, served as control. SDS was added to the 200 mL culture under magnetic stirring and air bubbled for 30 minutes. The foam formed on the surface was collected every 10 minutes with the help of a spatula and checked under the microscope to assess presence of nematodes and ciliates vestiges. When no more foam was formed, the air-bubbling was stopped and diatoms in the liquid observed through an inverted microscope to check cell viability and for the eventual presence of grazers. When grazers were still present the sample was further bubbled until they disappeared. Both treatments were tested in triplicate. Beckers with diatoms were stored in CT-room at 20° C, under a 16/8 h L/D (light/dark) regime at 100 μmol photon m^-2^ s^-1^ until the next day when MPB diatoms were inoculated on the hydrogel. Isolated diatom assemblage moving in a KC gel, was recorded using a digital optical microscope (VHX-7000, Keyence) into a short video clip using Da Vinci Resolve v.18.6 software.

In the second method, 10mL of 1.5% KC hydrogel polymerized in a 11 cm Petri dish, was laid on the top of the sediment and left for 24 hours to allow diatoms attaching to its surface. The hydrogel was transferred in its original Petri dish mould, covered with F/2 medium, and closed to avoid exsiccation.

### 2.6 Diatoms growth comparison in liquid and inside the hydrogel

Once the best hydrogel was selected, *N*. *spathulata*, *G*. *limosum*, *N*. *phyllepta*, growth in hydrogels and in liquid media were compared by inoculating the same biomass in 24-well plates in liquid and in 400 μL 1.5% KC hydrogels, both in triplicates. 50 μL of ultrapure water were added every two days until the end of the experiment to compensate for evaporation. Cell growth was followed through an inverted microscope (Olympus CKX53, 100X), on alternate days. Images were acquired through a microscopy camera (Axiocam, Zeiss) and cells were counted with ImageJ creating a macro with each cell specifics, i.e., size and sphericity [39].

### 2.7 Statistical analysis

Significant differences among the means of stiffness, water content and transparency of different hydrogels were tested with a one-way analysis of variance (ANOVA), followed by Tukey’s post-hoc test. Growth curve data distribution was tested using the Shapiro-Wilk normality test. Significant differences among the growth in liquid and hydrogel at each time point of the growth curves, were estimated by two-way ANOVA with Šídák post-hoc analysis for multiple comparisons. Levels of significance were set at *p* < 0.05; all statistical analyses were performed using GraphPad Prism, Version 6 (GraphPad Software, San Diego, CA, United States).

## 3. Results

### 3.1 Hydrogels screening

No synthetic PAAm-based hydrogel was suitable for diatom survival and growth, with water rinsing not being enough to flush out all the toxic compounds. All natural hydrogels: gelatin/Na-alginate, KC and Na-alginate hydrogels resulted in cell survival but presented some differences. Namely, Na-alginate hydrogels were instable in seawater after one week and gelatin/Na-alginate hydrogels promoted diatom adhesion on their surface, thus impairing free movement. A summary of the screening is presented in Table 1.

**Table 1:** Hydrogels screening for diatom inoculation. Columns report hydrogel tested chemical composition, observed cytocompatibility, mean ± SD of water content (%), stiffness values (kPa) and macroscopic observations.

Hydrogel tested	Cytocompatibility	Water content	Stiffness (kPa)	Observations
**PAAm-based**	Cells dead after few hours	81%±0.57	8.258±0.25	Yellowish color and difficult imaging
**PAAm-based + 2% Na-alginate**	Cells dead after few hours	88%±0.48	2.375±0.03	Yellowish color and difficult imaging
**Na silicate**	Cells dead after 1 day	65%±2.35	202.4±48.1	White translucent hydrogel
**Hyaluronic acid gel**	Cells vital up to two days without motility	93%±n.d.	0.205±n.d.	White transparent hydrogel
**Gelatin/ Na-alginate**	Long term survival with reduced motility	94.88%±0.04	0.152±0.02	Yellowish and transparent
**2% Na-alginate**	Long term survival and motility	98.61%±0.14	6.622±0.84	Transparent
**1.5% KC**	Long term survival and motility	98.43.%±0.11	6.921±0.69	Transparent

In PAAm hydrogels diatoms did not survive when the inoculum was done in the gel mix prior to polymerization and neither when cells were inoculated from the top after polymerization. Imaging the cells was also difficult due to the hydrogel high opacity.

The Na-silicate hydrogel tended to form silica precipitates due to small variations in the HCl volume added for gelation. The addition of 180 μL of HCl 4N induced hydrogel formation and pH lowering from 12 to 8. These hydrogels had a good translucent appearance, but diatoms died after one day and were not further investigated.

Modified hyaluronic acid gels had a white appearance but were transparent enough to allow cells observation with a microscope in transmittance. However, after two days, cells stopped moving and they did not divide.

**Fig 1:**
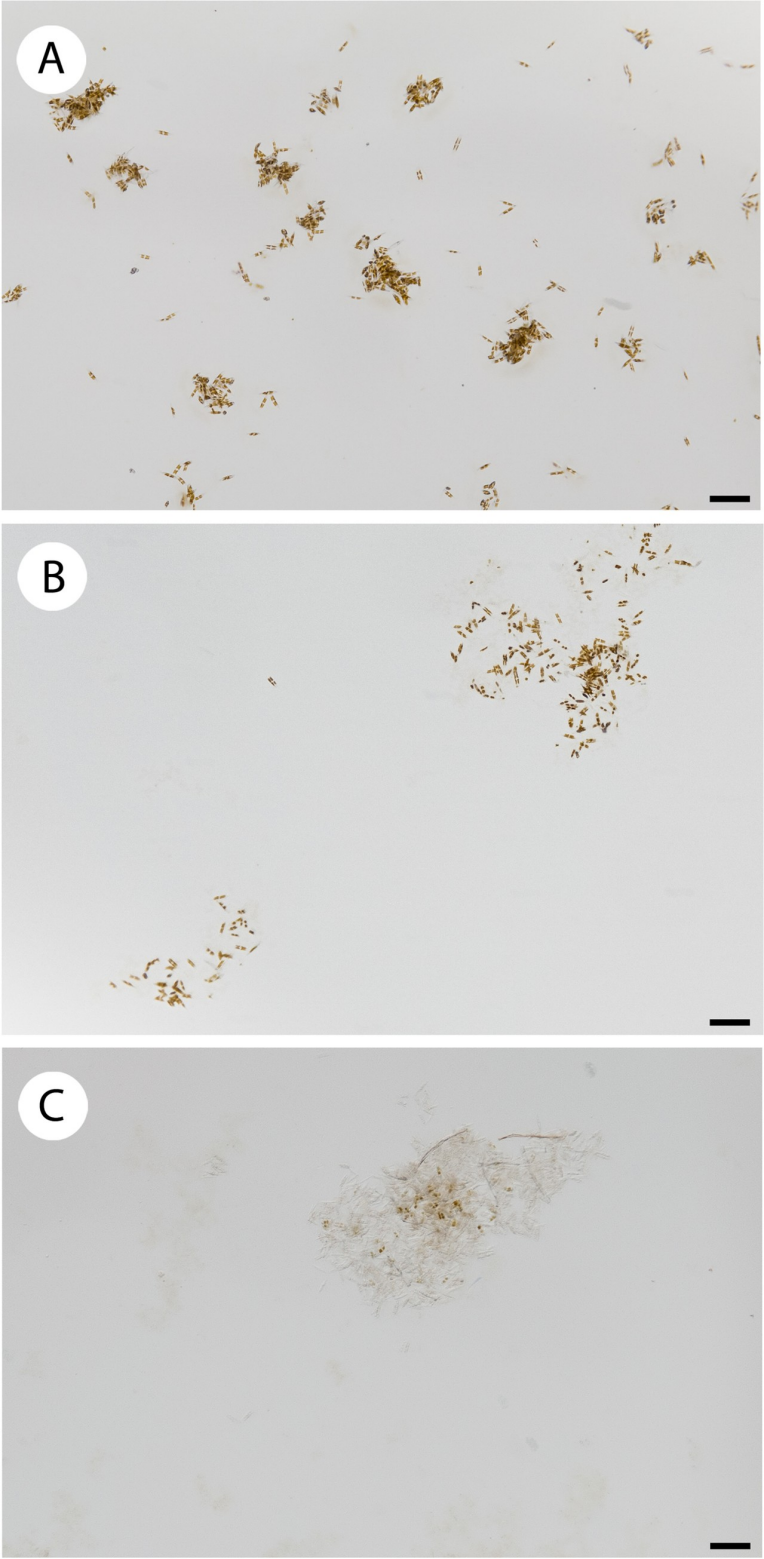
Diatoms different spatial distribution in 6%, 8%, 10% gelatin hydrogels. In 6% cells separate one from each other spreading in the gel (A); in 8% few cells move from the aggregate (B); in 10% cells remain aggregated in the inoculum point until dying (C). Scale bar = 100 μm.

The gelatin gels were too sticky and prevented diatoms from moving freely. The only concentration that allowed for diatom movement after 24h was 6% (Fig 1A). While, in 8% diatoms showed some movement and in 10% no movement was observed (Fig 1B and 1C).

**Fig 2:**
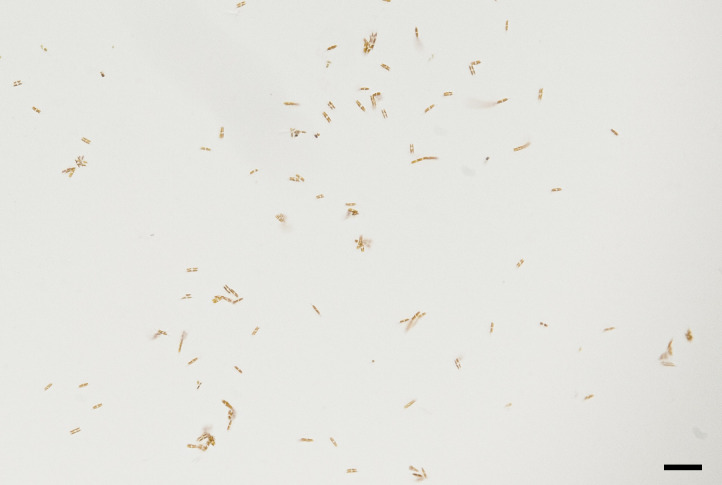
Diatoms in 2:1 1.5% Na-alginate: 8% gelatin hydrogel. Diatoms spread from the inoculum under the hydrogel surface. Scale bar = 100 μm.

In both higher gelatin concentrations, the diatoms died after 48 hours without spreading away from the inoculum point. However, in the 6% cells resisted up to one week slowly moving inside the gel and dispersing without remaining aggregated as in the 8% and 10% (Fig 1). In the mixed 1.5% Na-alginate/8% gelatin hydrogels the 1:2 ratio resulted in a sticky hydrogel while the 3:1 ratio produced a turbid gel. Only the 2:1, 1.5% Na-alginate: 8% gelatin hydrogel ratio, allowed diatom movement and cell proliferation inside the hydrogel, without aggregate formation (Fig 2).

The same result was observed adding the inoculum before the gel polymerization. The gelatin/ Na-alginate gel was 90% transparent with 94.88% ± 0.05 water content. Cells took more time to spread from the starting inoculum and stopped moving after four days.

The 2% Na-alginate gels cross-linked in 1% CaCl_2_ allowed diatoms growth and movement inside the hydrogel; both with the inoculum in the mix prior polymerization and the inoculum added from the surface. Cells moved similarly to what was observed in liquid medium for the same species. They were also observed moving in the vertical direction. Diatom movement was observed up to seven days for most cells, which is comparable to what is usually observed in liquid batch cultures. These gels were 88% transparent with high water content, 98.61% ± 0.14. After ten days of suspension in the F/2 medium the hydrogel started to degrade and to get opaque.

No differences were noticed between the inoculum from the top and in the gel mix prior polymerization in 2% Na-alginate. Diatoms mixed through the entire depth of the hydrogel started to move after 12 hours of acclimatization and they colonized the entire hydrogel in the same way.

KC hydrogels were the easiest to fabricate, with polymerization occurring within five minutes. They were 92% transparent with high water content 98.43% ± 0.11. Diatoms survived in these gels for up to 2 months with the gels showing almost no visible degradation. Diatoms kept motility for 7 days, similarly to what was observed in the liquid batch cultures.

No link was found between hydrogels stiffness (storage modulus) and diatom movement. Except for Na silicate gels that showed significantly higher storage modulus values (*p* < 0.0001), no other significant differences in storage modulus values were observed between the different gels.

Water content was significantly different between most gels (*p* < 0.005). The exceptions were between gelatin/Na-alginate and MeHA (*p* = 0.2422) and between Na-alginate and KC (*p* > 0.99). The lowest EWC values were found in Na silicate (65% ± 2.35) gels.

Transparency values were less variable between gels with the only significant difference being observed between KC and Na-alginate gels (*p* < 0.05), with higher transparency in KC.

### 3.2 Diatoms transfer from the MPB to the hydrogel

**Fig 3:**
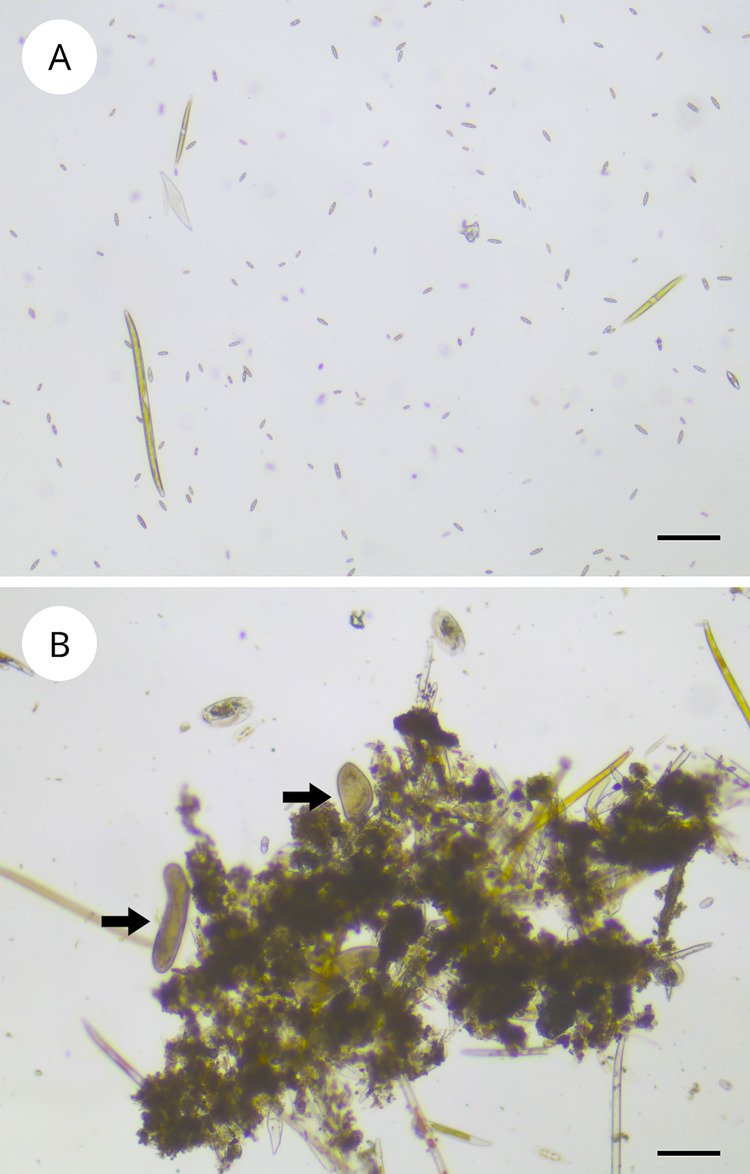
Effect of sodium dodecyl sulphate on grazers elimination from the microphytobenthos. Diatoms isolated from the microphytobenthos after sodium dodecyl sulphate treatment at 50 mg/L (A) and the control (B). Black arrows indicate grazers. Scale bar = 100 μm.

Diatoms extracted from the sediment with the lens tissue method could be kept alive in liquid medium for one week maximum before nematodes and ciliates prevailed in the culture. To keep MPB diatom assemblages alive for longer out of the sediment we tried adding 50 mg/L SDS to 200 mL MPB culture attempting to eliminate meio- and micro-fauna. This treatment successfully eliminated all ciliates and nematodes (Fig 3A). The control without SDS showed the abundancy of microorganisms normally present in the natural MPB (Fig 3B).

**Fig 4:**
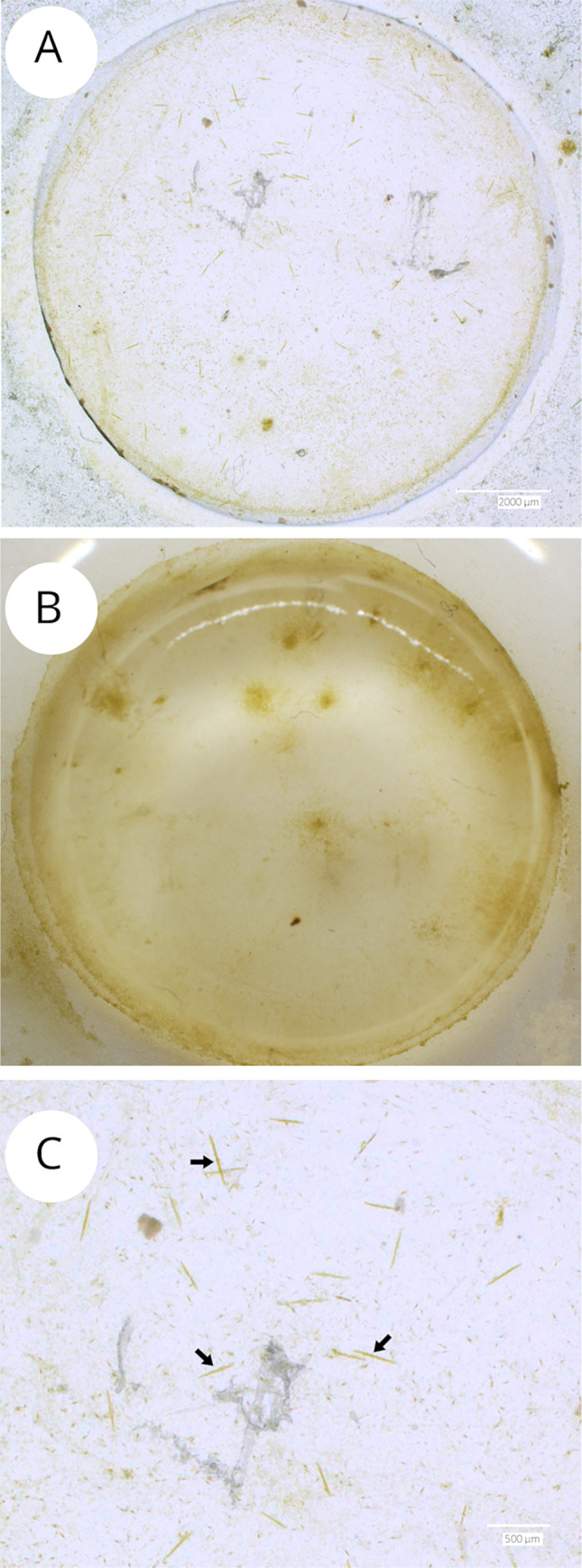
Kappa-carrageenan (KC) hydrogel with microphytobenthos cleaned with sodium dodecyl sulphate maintained in culture over 60 days. KC hydrogel with isolated natural microphytobenthos after 20 days from the inoculum (A) and 60 days (B); KC hydrogel (A) detail of *Nitzschia elongata* cells among other unidentified diatoms (C).

Consequentially, the isolated MPB was inoculated in KC hydrogel which was kept up to two months (Fig 4) with diatoms keeping moving and growing during this time (S1 Video). This condition also allowed larger size cells such as *Nitzschia elongata* to survive and grow (Fig 4C), which is something we were never able to achieve in liquid medium.

Additionally, we tried transferring MPB assemblages directly into the 1.5% KC hydrogel by depositing the gel on top of the sediment for 24 hours, allowing diatoms to move into the gel along with ciliates and nematodes. However, within one week, the grazers had overgrown the diatoms impeding long-term maintenance of the hydrogel (> 1 week).

### 3.3 Diatoms growth comparison in liquid and inside the hydrogel

**Fig 5:**
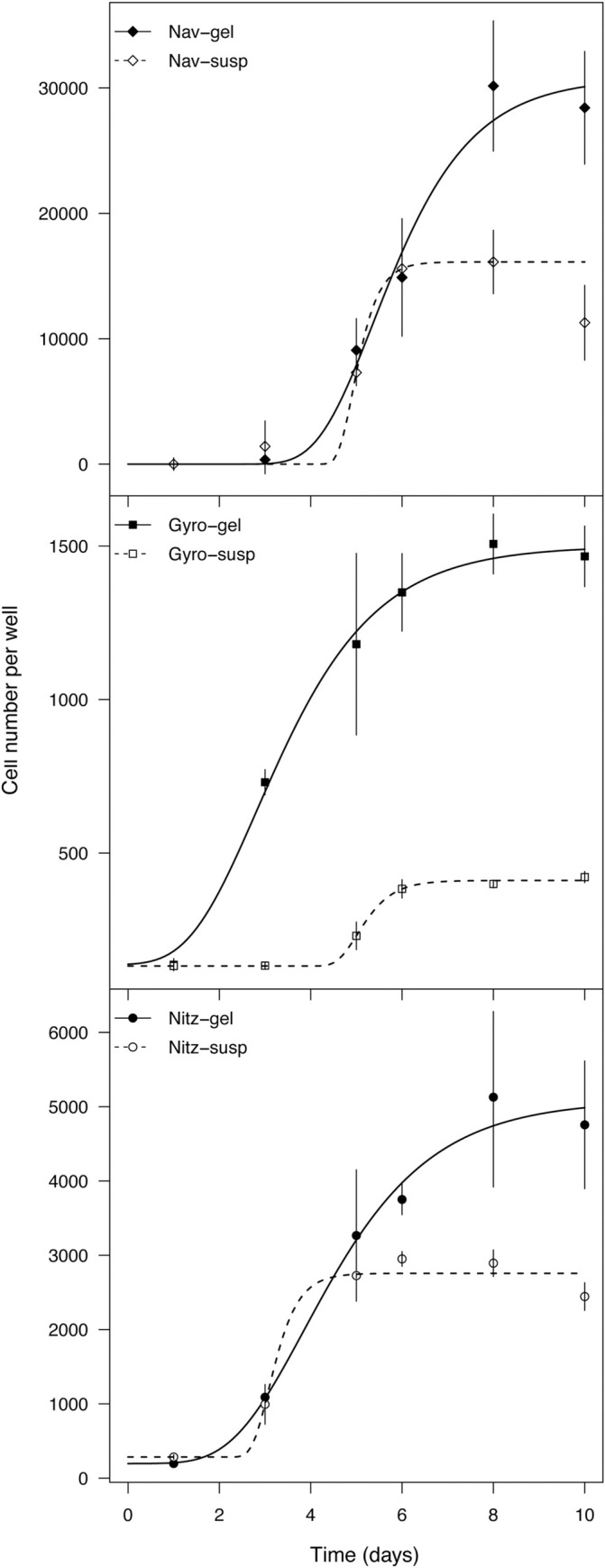
*Navicula phyllepta*, *Gyrosigma limosum*, *Nitzschia spathulata* growth curves in kappa-carrageenan hydrogel and in suspension over 10 days. Error bars are standard deviations of the means, n = 3.

Diatom growth in hydrogels performed better than in liquid medium for all the three tested diatoms (Fig 5).

*Navicula phyllepta* showed twofold higher (*p* <0.0001) final biomass yields in the stationary phase in hydrogels in comparison to the liquid condition (day 8 and 10); the lag phase and the exponential showed non-significant differences between liquid and hydrogel (from day 1 to 6). *Nitzschia spathulata* also showed no significant differences in the exponential phase between liquid and gel conditions; approximately twofold (1.77) higher biomass yields were also obtained in gels at the stationary phase. *Gyrosigma limosum* grew slower in liquid medium, with a short exponential phase and lower increase in cell number, while in growth in hydrogels showed four days of exponential phase and fourfold higher cell numbers.

## 4. Discussion

In this work we provided a tool to study the diatoms vertical migration in controlled conditions, fabricating transparent, cytocompatible hydrogels. The hydrogel allows direct measurements of the species composition and cell movement, without recurring to indirect methods. Different hydrogels never tested before for algae embedding were screened and KC hydrogels were selected as the best to study benthic diatom movement and to keep viable cells for up to 2 months. Three possible methods to study diatoms from MPB with hydrogels were tested: 1) single cell isolation and reconstruction of natural MPB assemblages; 2) microphytobenthic diatom isolation from nematodes and ciliates and inoculation in hydrogel; and 3) MPB population transfer on hydrogels. Finally, the growth of three diatom species was compared between liquid F/2 medium and hydrogels, showing higher performances in the latter.

### 4.1 Hydrogels properties

None of the synthetic hydrogels tested (PAAm, PAAm-alginate, Na-silicate) allowed diatom survival, differently from the natural ones (gelatin, Na-alginate, KC). Natural hydrogels have the advantage of being more biocompatible than synthetic, but they have drawbacks. Namely, they are more biodegradable and show higher batch-to-batch variation, which affects the repeatability [40]. For this reason, modified natural hydrogels, like MeHA in our study, with increased stability and more complex polymer composition can be constructed by combination with synthetic molecules, to mild both synthetic and natural hydrogels properties [41]. However, despite synthetic hydrogels can be better controlled and more finely tuned, they might be more cytotoxic depending on the type of cells embedded [36, 42]. Due to the several variables in hydrogels composition and properties, together with the embedded cells physiological characteristics, it is difficult to determine a priori a suitable hydrogel to grow cells in. The first requirement to fill is the hydrogel cytocompatibility. Cytocompatible hydrogels are sought for medical applications, they must be non-toxic and not give side effects, generally with reference to animal tissues [43]. Relevant information on cytotoxicity can be found in the product’s safety data sheet. Section 12 of the safety data sheet regards the environmental impact (regulation EU No 2020/878), here any hazardous effects on microalgae should be reported [44]. However, information on phytotoxicity is not mandatory, so toxicity data on microalgae can be not available.

The Polyamidoamine-based hydrogels exist in a linear form (PAA) and a dendrimer-like form (PAMAM). They are both versatile synthetic hydrogels, mainly used in biotechnologies as drug carriers [45]. The PAA hydrogels we tested, were lethal for diatoms in both the classic formulation and with the Na-alginate addition. Rinsing and lyophilization did not lead to any improvement. They were therefore excluded from further analysis. PAA phycotoxicity has not been reported so far, the only available information on cytotoxicity regards human cells applications, where no side effects have been found [46, 47]. In the Polyamidoamine dendrimer-like form instead, (PAMAM) toxicity was tested on cells of *Chlamidomonas reinhardtii* resulting toxic at a concentration of 2.5 mg L^−1^ [48].

Benthic diatoms in Na-silicate hydrogels resulted in only one day of vitality, most likely because of further pH changes after gelation which could not be detected by the pH strips [49]. In addition to cells dying on the hydrogel surface, the much higher measured G values (202.4 ± 48.1 Kpa) could have hindered diatom movement below the surface, being 25 times higher than the Na-alginate and the KC hydrogels. Different studies showed microalgae responses to different hydrogel stiffnesses cannot be compared being species-specific and the silica gel properties need to be tuned accordingly [50–52]. For instance, Na-silicate hydrogels were successfully used for *C*. *reinhardtii* growth [53] and in testing diatom ability for taking up silica from the hydrogel matrix for building their frustule [54]. In this study [54] authors obtained positive results with *Phaeodactylum tricornutum* and *Cylindrotheca fusiformis* in Na-silicate but not with centric diatoms such as *Coscinodiscus sp*. Studies on *Chlamydomonas* cells entrapment in Na-silicate screened different formulations to reduce silica hydrogel stiffness and to promote cell proliferation, demonstrating for the first time that the hydrogel stiffness was limiting cell viability [49, 53]. Measuring the hydrogels rheological properties and the embedded diatoms response, could be therefore important for benthic diatoms inhabiting mudflat sediment with variable stiffness.

Hyaluronic acid (HA) is a natural polysaccharide found in the extracellular matrix of all animal tissues and HA hydrogels are the most used material in cosmetic surgery [55, 56]. Once chemically modified by methacrylation, like in this study, the MeHA gel is considered suitable to be crosslinked with the SH group coming from the DTT, using a Michael addition reaction [57]. This modification increases gel stiffness and stability, whilst simultaneously being biocompatible for human applications [58]. To our knowledge, microalgae embedded in methacrylate hyaluronic acid hydrogels have never been tested but combining this type of hydrogel with microalgae has been proposed for oxygen supply in implants [59]. In our work, diatoms remained alive for only two days. No further tests were conducted in improving cell viability, our main consideration being the high cost of hyaluronic acid in comparison to kappa carrageenan (KC). However, for algae embedding in human prosthesis, the HA hydrogel suitability for algae growth can be further developed by trying different HA molecular weights and changing of crosslinking methods. In human cells applications it has been shown that different molecular weights can trigger different biological responses [60].

Gelatin is a protein-based natural polymer that has been widely used in food, pharmaceutical and cosmetics industries and laboratories [31, 61–63]. Microalgae embedded in gelatin hydrogel have been already used for human consumption (patented by Solazyme, Inc.) [64]. However, gelatin has recently lost interest in food applications since is derived from animal products, and it is being mainly substituted by vegetal alternatives like Na-alginate or carrageenan [65, 66]. Thanks to its high bioadhesive properties, gelatin is more suitable for cells immobilization than movement. In gelatin 8% and 10% cells formed aggregates (Fig 1B and 1C) since they were not able to move after the inoculum and they did not spread. Even if we could observe movement in the 6% gelatin hydrogel, *Nitzschia spathulata* cells did not migrate far from the inoculum point and they stopped moving after few days without colonizing the entire gel. To modify the bio-adhesive property of gelatin hydrogels and to facilitate cell motility inside the gel, Na-alginate was introduced to form gelatin/Na-alginate hydrogel.

The 2% Na-alginate gels showed similar characteristics as the KC hydrogel in terms of transparency, EWC, stiffness, reflected also in the diatoms similar behaviour inside these two hydrogels. The only drawback was the low stability of Na-alginate in F/2 medium, probably due to the ions naturally present in the seawater, reducing the gel stability to 7 days [67].

The KC hydrogel was 92% transparent, was stiff enough (6.921±0.69 kPa) to keep its stability and prevent the cell leakage. The gel maintained its characteristics in terms of stability and transparency up to two months in F/2 medium.

In gelatin/Na-alginate, Na-alginate and KC hydrogels diatoms could grow, move, and migrate inside the gels. The three hydrogels were transparent enough (from 88% to 92%) to allow light penetration for growth and for directly observing the cells in transmittance optical microscopy. In addition, they all had the soft texture necessary for simulating MPB migration in the natural sediments, hence allowing benthic diatoms vertical movement to be studied in these substrates. As a reference, mudflats Storage modulus measured in [68] was around 10 kPa at ~1350 kg/m^3^ density at the mudflat surface, where the biofilm is more concentrated, which is comparable to Na-alginate (6.622 kPa) and KC (6.921 kPa) hydrogels stiffness.

Hydrogels high water content also allowed diatoms to keep their natural movement, which was especially high in gelatin/Na-alginate, Na-alginate and KC (from 95% to 99%). The lowest EWC values were found in Na silicate (65%±2.35) gels which was still comparable to mudflat EWC measured in [69, 70], which observed values between ~70% and ~62% respectively, with variations according to the sites and seasons. Based on these characteristics all the three hydrogels were suitable to grow diatoms and investigate MPB vertical movements. However, the observations based on hydrogel stability and diatom movement led our choice towards the KC gel.

### 4.2 Microphytobenthic assemblages in hydrogels

Benthic diatoms seem to need a substrate to adhere for their growth [71]. Although some species can grow in suspension like *Cylindrotheca closterium* [68, 72], others like *Gyrosigma limosum* form a biofilm on the bottom and they do not seem to grow in suspension (pers. obs.). Hydrogels can provide a support for growing cells and facilitate the isolation of benthic diatoms when they do not in liquid, e.g., the two *Nitzschia* species in our study. Many studies have been conducted on freshly collected MPB [6, 7, 11, 18, 72–75], mainly due to the difficulty of isolating single species and of keeping the MPB assemblages viable for long time scales in laboratory conditions. Embedding diatoms in hydrogels allows MPB natural assemblages to colonize the gels within a 3D matrix where potentially different species can move to different depths and orientate differently towards light sources, similarly to what has been observed in natural sediments [2, 4, 6–11, 19, 74, 76, 77]. This study tested three methods to observe MPB diatom movement outside mudflat sediments: 1) isolation of single species from sediments and inoculation in hydrogels; 2) elimination of ciliates and nematodes from MPB assemblages; 3) transference of fresh MPB assemblages and associated grazers onto hydrogels surface.

Our hydrogel system allowed the isolation of large benthic species which did not grow in liquid medium (e.g., *Nitzschia elongata*). This technique could be used to isolate other large pennate diatoms which are not found in the algae banks. Isolating the main species composing the MPB, can help to recreate the natural population according to the taxonomic data available relative to a specific sampling site [78]. Once new strain cultures are set up in laboratory conditions, they can be inoculated in the hydrogel where different light conditions can be tested, and their movement followed by imaging techniques. This can extend the knowledge on the photoregulation system in diatoms by controlling their growth parameters and light history once the cultures are established in laboratory conditions. Information on photoregulation is not available for many benthic species even if they are highly represented in the MPB and which could be of high interest due to their complex photoregulative behaviour [10, 73]. The availability of these benthic diatoms in culture could be used not only to observe their movement against the light in the hydrogel but also to investigate the genes involved in this mechanism [79].

Studies on the use of SDS to obtain diatoms assemblages free from the grazers have not been conducted yet. In this study we used this method to get rid of the meiofauna normally present in the freshly collected MPB, which could be used to isolate single species, to observe their endogenic rhythms, to change the abiotic conditions and observe their response to stimula (e.g., by variable fluorescence analysis). SDS is a surfactant whose principle is the separation of solutions with different charges, including membranes [80]. Surfactants can facilitate the separation of algae from water by rendering surfaces more hydrophobic [81]. However, SDS alone is not efficient to bind microalgae membranes, being an anionic collector, it needs to be combined with other surfactants or to work at low pH (5.0–8.0) [82]. Under these conditions, SDS surfactant property has been exploited for harvesting microalgae by flotation and also for contaminant removal, e.g. ciliates from *C*. *vulgaris* [83, 84]. Using this technique on diatoms has the advantage of the silica frustule protecting the diatom cell membrane from the SDS action, and to bind instead to the ciliates and nematodes membranes. Diatoms stopped moving immediately after SDS use, but they recovered motility after one day of incubation in the CT-room, and they could be inoculated in a KC hydrogel where they lasted until 2 months. As for *N*. *spathulata* isolation and growth, *N*. *elongata* inoculated from the MPB, started to reproduce and grow in the hydrogel, while it did not grow when isolated in liquid (Fig 4C).

In the third method, the MPB population was transferred on the KC hydrogel laid on the mudflat surface. Diatoms and grazers transferred on the hydrogel, could move and proliferate inside the hydrogel, passing from the surface to deeper inside the gels. Due to the grazers overgrowth, experiments using this technique can last around one week depending on grazers proliferation. Bott et al., (1999) [85] reported meiofauna ingestion rate of 1.6% of diatoms abundance per hours, which would reach the 100% in 2.6 days, which is even less that what we have observed (~one week). Different studies demonstrated grazers preference for large diatoms which highlights the importance of isolating large size diatoms to rebuild the MPB community in laboratory conditions [86, 87]. Occasionally overgrazing has been observed with grazing rates exceeding the MPB productivity [84, 85]. Even if this was not investigated in our study, grazers interaction with the MPB can be observed through the proposed system, for example species preferences and abundance over time in hydrogel controlled conditions.

### 4.3 Benthic diatom growth in liquid medium vs hydrogel

Previous studies showed that microalgae growth could be improved by using hydrogels [32, 88]. These studies focused on microalgae growth trends in hydrogels and liquid and their observations are similar to the ones made in our study: 1) non-significant difference between free cells and immobilized cells growth in the exponential phase, therefore showing similar growth rates; 2) higher final biomass in hydrogel; this is more predictable in our case since benthic diatoms do not grow in suspension and growing in a 3D space increases the surface available to colonize, resulting in a higher number of cells compared to a 2D space; 3) longer lag phase in hydrogel, not observed in our study, probably due to the already lower growth rate of the large species used in this study, compared with fast growing species like *Chlorella* where the difference is more noticeable; 4) higher photosynthetic activity in hydrogel (not investigated in this study). Just few studies tested microalgae embedment in KC, for growth improvement, wastewater treatments and bioprinting [32, 89, 90]. In one of these studies [32], *Chlorella vulgaris* was cultivated and immobilized in KC 2.5%, where it showed similar growth than the control in liquid but exhibited higher photosynthetic activity. This last characteristic has been explained by the authors, through a compensation for immobilized cells shading in hydrogel which enhanced chlorophyll accumulation in the cells to compensate for receiving less light and for the lack of motility [32].

## 5. Conclusions

Our novel strategy to study the MPB in hydrogel and in controlled conditions resulted in a straightforward technique to investigate species-specific photoregulation of benthic diatoms. This can encourage further investigations to fill the missing information on single species response to light and to unravel the mechanisms regulating their endogenic rhythms. The isolation of new strains and of the entire MPB assemblage with and without grazers can provide insights into the complex relationships between these organisms under controlled conditions.

## Supporting information

S1 Video Microphytobenthic diatom assemblage moving in kappa-carrageenan hydrogel in real time. (MOV)
